# Efficacy of TB-PCR using EBUS-TBNA samples in patients with intrathoracic granulomatous lymphadenopathy

**DOI:** 10.1186/s12890-015-0162-4

**Published:** 2015-12-28

**Authors:** Jung Seop Eom, Jeong Ha Mok, Min Ki Lee, Kwangha Lee, Min Ji Kim, Sun Mi Jang, Hae Jung Na, Seung Eon Song, Geewon Lee, Eun-Jung Jo, Mi-Hyun Kim, Ki Uk Kim, Hye-Kyung Park

**Affiliations:** Department of Internal Medicine, Pusan National University School of Medicine, 179 Gudeok-ro, Seo-gu, Busan, 602-739 Korea; Department of Radiology, Pusan National University School of Medicine, Busan, Korea; Biomedical Research Institute, Pusan National University Hospital, Busan, Korea

**Keywords:** Endobronchial ultrasound-guided transbronchial needle aspiration, Polymerase chain reaction, Sarcoidosis, Sensitivity and specificity, Tuberculous lymphadenitis

## Abstract

**Background:**

Endobronchial ultrasound-guided transbronchial needle aspiration (EBUS-TBNA) is widely used to perform mediastinal lymph node sampling. However, little information is available on polymerase chain reaction for *Mycobacterium tuberculosis* (TB-PCR) using EBUS-TBNA samples in patients with intrathoracic granulomatous lymphadenopathy (IGL).

**Methods:**

A retrospective study using a prospectively collected database was performed from January 2010 to December 2014 to evaluate the efficacy of the TB-PCR test using EBUS-TBNA samples in patients with IGL. During the study period, 87 consecutive patients with isolated intrathoracic lymphadenopathy who received EBUS-TBNA were registered and 46 patients with IGL were included.

**Results:**

Of the 46 patients with IGL, tuberculous lymphadenitis and sarcoidosis were diagnosed in 16 and 30 patients, respectively. The sensitivity, specificity, positive predictive value, and negative predictive value of TB-PCR for tuberculous lymphadenitis were 56, 100, 100, and 81 %, respectively. The overall diagnostic accuracy of TB-PCR for tuberculous lymphadenitis was 85 %. In addition, seven (17 %) patients had non-diagnostic results from a histological examination and all of them had non-diagnostic microbiological results of an acid-fast bacilli smear and culture. Four (57 %) of the seven patients with non-diagnostic results had positive TB-PCR results, and anti-tuberculosis treatment led to clinical and radiological improvement in all of the patients.

**Conclusions:**

TB-PCR using EBUS-TBNA samples is a useful laboratory test for diagnosing IGL. Moreover, this technique can prevent further invasive evaluation in patients whose histological and microbiological tests are non-diagnostic.

## Background

Intrathoracic granulomatous lymphadenopathies (IGLs), such as tuberculous lymphadenitis and sarcoidosis, are frequently encountered by respiratory physicians, and their diagnosis is based on histological and microbiological tests [1–3]. Conventional transbronchial needle aspiration or mediastinoscopy has traditionally been used to perform lymph node biopsy for histological examinations, as well as for stains and cultures for acid-fast organisms [4–6]. With the recent advent of endobronchial ultrasound, endobronchial ultrasound-guided transbronchial needle aspiration (EBUS-TBNA) has been widely used to perform mediastinal lymph node biopsy or aspiration. There is increasing evidence regarding EBUS-TBNA as the first examination of choice in the evaluation of patients with IGL [7–9].

Most IGLs comprise tuberculous lymphadenitis and sarcoidosis. Occasionally, differentiating between these IGLs using a histological examination alone is difficult. Moreover, the diagnostic yield of acid-fast bacilli culture is still unsatisfactory, although such culture is considered the gold standard diagnostic method for tuberculosis. In addition to histological and microbiological tests, polymerase chain reaction for *Mycobacterium tuberculosis* (TB-PCR) is recognized as a useful test in the differential diagnosis of tuberculous lymphadenitis and sarcoidosis [10]. However, little information is available on TB-PCR using EBUS-TBNA samples. Therefore, we conducted this study to examine the diagnostic performance of TB-PCR using EBUS-TBNA samples in patients with IGL. We analyzed a prospectively collected database in South Korea, where the incidence of tuberculosis is intermediate (97/100,000 per year) [11].

## Methods

### Study population

**Fig. 1:**
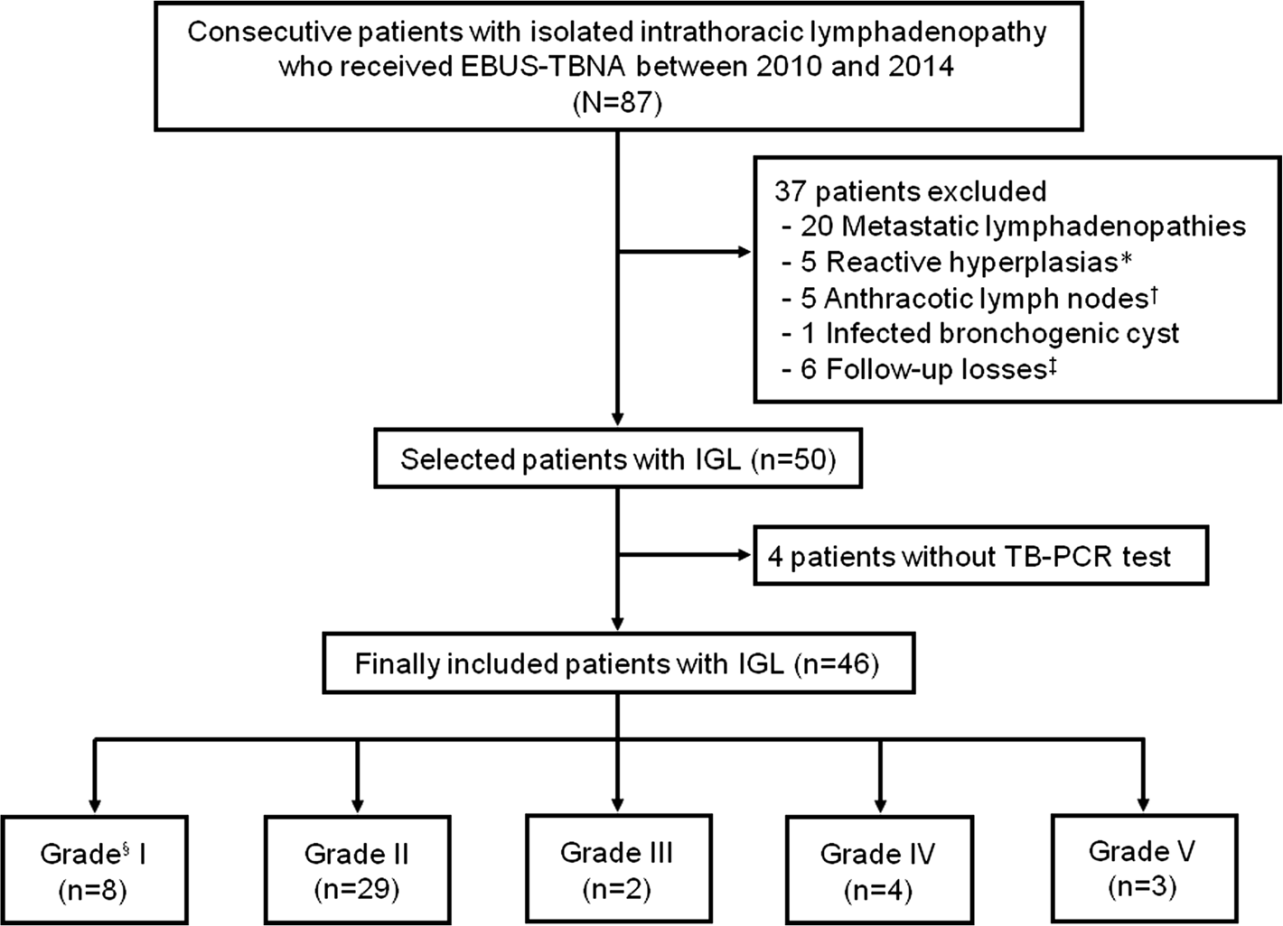
Study flow diagram. *Of the five patients with reactive hyperplasia, two were confirmed by subsequent mediastinoscopy, and a CT scan of the remaining three patients showed decreased or unchanged lymph node sizes. †All five patients with anthracotic lymph nodes were followed up for more than 6 months, and the lymph node size was decreased or unchanged on subsequent CT. ‡In six patients who were lost to follow-up, the results of EBUS-TBNA were insufficient specimens in three patients and reactive hyperplasia in the other patients. §Histological specimens were classified into five grades: I) epithelioid granulomatous reaction with caseation, II) epithelioid granulomatous reaction without caseation, III) nongranulomatous reaction with necrosis, IV) nonspecific, and V) inadequate sample. EBUS-TBNA, endobronchial ultrasound-guided transbronchial needle aspiration; IGL, intrathoracic granulomatous lymphadenopathy; TB-PCR, polymerase chain reaction for *Mycobacterium tuberculosis*

From January 2010 to December 2014, a retrospective study with a prospectively collected database was performed to evaluate the efficacy of TB-PCR using EBUS-TBNA samples in patients with IGL at Pusan National University Hospital (university-affiliated, tertiary referral hospital in Busan, South Korea). During the study period, all consecutive patients who received EBUS-TBNA were prospectively registered. As a result, 87 patients with isolated intrathoracic lymphadenopathy, defined as lymphadenopathy without lung parenchymal abnormalities, received EBUS-TBNA. Fifty patients were diagnosed with IGL (Fig. 1). Of the 50 selected patients with IGL, four who were not subjected to TB-PCR were excluded from the analysis. Therefore, 46 patients with IGL were finally included in the present study.

The Institutional Review Board of Pusan National University Hospital approved this study (No. E-2015040). Informed consent was waived because of the retrospective nature of the study.

### EBUS-TBNA procedure

All bronchoscopic procedures were performed under conscious sedation with local anesthesia by three pulmonary physicians (Eom JS, Mok JH, and Lee K). Before EBUS-TBNA, conventional bronchoscopy was conducted in a standard fashion for airway inspection and administration of lidocaine into the tracheobronchial tree via the working channel of the bronchoscope. Following conventional bronchoscopy, EBUS-TBNA was performed using a dedicated bronchoscope with a linear ultrasound transducer (BF-UC260F-OL8; Olympus, Tokyo, Japan). Systemic assessment of the mediastinal, hilar, and interlobar lymph nodes was made based on computed tomography (CT) findings [12], and target lymph nodes were punctured with a 22-gauge needle (NA-201SX-4022; Olympus, Tokyo, Japan) under real-time ultrasound guidance. Two or more punctures were performed on each target lymph node until at least two tissue core specimens were obtained. One of the tissue core specimens was placed in formalin for histological examination. Other tissue core specimens that were placed in sterile saline were analyzed by fluorescence microscopy using auramine-rhodamine staining. Additionally, solid (3 % Ogawa medium) and liquid media (BacT/ALERT MP; bioMérieux, Durham, NC, USA) were used to culture for mycobacteria from the specimens in sterile saline.

### TB-PCR

Nested PCR (for formalin-fixed paraffin-embedded specimens) or real-time PCR (for specimens in sterile saline) was conducted to detect nucleic acids of *M. tuberculosis* using tissue core samples collected by EBUS-TBNA.

For nested PCR, formalin-fixed paraffin-embedded tissues (12-μm thick) were incubated in 1 mL of xylene at 60 °C for 30 min and then centrifuged for 10 min (8800 rpm). Paraffin and the supernatant were removed from the samples after centrifugation. The same procedures were repeated until deparaffinization was complete. After adding 1 mL of alcohol, the samples were centrifuged for 5 min (8800 rpm), and the supernatant was removed. The samples were then air-dried as pellets. DNA was extracted using QIAamp (Qiagen, Valencia, CA, USA) according to the manufacturer’s instructions. PCR amplifications were performed using *M. tuberculosis IS6110* primers (MTB-PCR kit; Biosewoom, Seoul, South Korea) according to the manufacturer’s instructions. The first round using outer primers and the second round using inner primers amplified a 256- and 181-bp fragment, respectively. Finally, the PCR products were visualized in a 2 % agarose gel.

For real-time PCR, specimens in sterile saline were filtered and 1 mL of phosphate-based saline was added. After centrifugation for 3 min (13,000 rpm), the supernatant was removed. Next, 50 μL of extraction buffer was added to the sample, and the sample was heated at 100 °C for 20 min. After centrifugation for 3 min (13,000 rpm), the supernatant was used in PCR. Real-time PCR was performed using the AdvanSure TB/NTM real-time PCR kit (LG Life Science, Seoul, South Korea) according to the manufacturer’s protocol. Three channels were used in the real-time PCR reaction (*M. tuberculosis* complex, mycobacteria, and internal control). Signals for FAM, HEX, and Cy5 were measured in each channel. *M. tuberculosis* was considered present if the cycle threshold of *rpoB* was less than 35 in each signal and greater than or equivalent to that of *IS6110*.

### Diagnosis of tuberculous lymphadenitis and sarcoidosis

Tuberculous lymphadenitis was regarded as present when *M. tuberculosis* was cultured in EBUS-TBNA samples. Patients with histological findings suggesting tuberculosis (granulomatous reaction with caseation necrosis or multinucleated giant cells associated with epithelioid histiocytes) or radiological findings compatible with tuberculous lymphadenitis (CT findings of nodes with central low attenuation and peripheral rim enhancement) were also considered to have tuberculosis only if clinical and radiological improvement was achieved after the standard anti-tuberculosis treatment [13]. Clinical and radiological improvement was defined as disappearance of symptoms and a decrease in the size of lymph nodes at a follow-up radiological examination. The diagnosis of sarcoidosis required compatible clinical and radiological results, exclusion of other granulomatous diseases with a similar histological or clinical picture (e.g., tuberculous lymphadenitis), and histological demonstration of noncaseating granulomas [1, 3].

### Classification of granulomatous inflammation

Histological specimens were classified into five grades as previously reported [14]: I) epithelioid granulomatous reaction with caseation, II) epithelioid granulomatous reaction without caseation, III) nongranulomatous reaction with necrosis, IV) nonspecific, and V) inadequate sample. If two or more lymph nodes were examined in one patient, the most suitable specimen for diagnosis was selected for analysis.

### Statistical analysis

Analyses were conducted on a per-patient basis according to the final diagnosis and results of the TB-PCR tests. All categorical and continuous variables are shown as numbers (percentages) and means (standard deviation), respectively. The overall diagnostic accuracy of TB-PCR for tuberculous lymphadenitis was calculated as follows: diagnostic accuracy (%) = number of patients with IGL who were accurately diagnosed (total number of true-positive and true-negative cases)/total number of patients with IGL [15]. *P* values of <0.05 were considered to indicate significance. All analyses were conducted using SPSS for Windows version 17.0 (SPSS Inc., Chicago, IL, USA).

## Results

### Study population

Using EBUS-TBNA, 82 lymph nodes were examined in 46 patients with IGL. All of the patients showed negative results using a screening assay for antibody to human immunodeficiency virus. Baseline characteristics are shown in Table 1. The mean age of the patients was 47.1 ± 17.1 years and there were 28 (61 %) female patients. The mean shortest diameter of lymph nodes that were examined using EBUS-TBNA was 17.7 ± 5.0 mm (median, 17.9 mm) in patients with sarcoidosis and 22.6 ± 11.3 mm (median, 20.0 mm) in patients with tuberculous lymphadenitis.

**Table 1:** Baseline characteristics of 46 patients with intrathoracic granulomatous lymphadenopathy

Variables	No. (%) or mean ± SD
Age	47.1 ± 17.1
Female gender	28 (61)
Number of LN involved per patient^a^	7.0 ± 3.6
Number of LN examined per patient	1.7 ± 0.7
Number of needle pass per LN	3.7 ± 1.8
Number of tissue core achieved per LN	2.0 ± 1.3
Stations of LN punctured^b^	
2R	2/82 (2)
4R	31/82 (38)
4 L	3/82 (4)
7	33/82 (40)
10R	1/82 (1)
11R	9/82 (11)
11 L	3/82 (4)
Diameter of LN examined using EBUS-TBNA^c^	
Shortest diameter, mm	19.4 ± 8.0
Longest diameter, mm	28.7 ± 11.4

^a^Involved lymph node was defined as the shortest diameter ≥ 1 cm on a CT scan

^b^Based on the International Association for the Study of Lung Cancer (IASLC) lymph node map

^c^Measured on an axial CT scan

SD, standard deviation; LN, lymph node; EBUS-TBNA, endobronchial ultrasound-guided transbronchial needle aspiration

### Diagnosis of tuberculous lymphadenitis and sarcoidosis

Of the 46 patients with IGL, 16 with tuberculous lymphadenitis and 30 with sarcoidosis were identified. Sputum acid-fast bacilli smear and *M. tuberculosis* culture were positive in 13 % (2/16) and 19 % (3/16) of patients with tuberculous lymphadenitis, respectively. According to the results of the *M. tuberculosis* culture, three patients had a confirmative diagnosis of tuberculous lymphadenitis. Based on the clinical and radiological responses after anti-tuberculosis treatment or histological results, the remaining 13 patients were diagnosed with tuberculous lymphadenitis. Of 30 patients with sarcoidosis, 28 were diagnosed according to compatible clinical, radiological, and laboratory results with demonstration of noncaseating granulomas using EBUS-TBNA samples. Mediastinoscopy was subsequently performed in one patient whose EBUS-TBNA was shown to be anthracotic pigmentation with inflammation (Patient 1 in Table 2). The surgical specimen showed chronic granulomatous inflammation without caseous necrosis, and sarcoidosis was finally diagnosed with compatible radiological and laboratory results. Another patient whose EBUS-TBNA samples were shown to be inadequate (grade V) refused mediastinoscopy. Therefore, she was followed up for more than 12 months and clinically diagnosed with sarcoidosis based on radiological and laboratory results (Patient 6 in Table 2).

**Table 2:** Detailed characteristics of seven patients with grade IV or V for histological results

Characteristics	Patient number						
	1	2	3	4	5	6	7
Sex	Female	Male	Male	Female	Female	Female	Female
Age, years	65	78	27	73	63	33	56
Number of LN involved per patient^a^	3	3	3	2	4	8	4
Number of LN examined per patient	1	2	1	2	2	2	1
Number of needle pass per LN	6	4 and 2	4	3 and 3	2 and 2	6 and 2	1
Number of tissue core achieved per LN	2	0 and 2	1	1 and 2	2 and 2	1 and 1	0
Stations of LN punctured	7	7 and 11R	4R	4R and 10R	2R and 4R	4R and 7	4R
Shortest diameter of LN punctured, mm	10.6	13.0 and 15.0	32.4	18.9 and 12.2	14.6 and 10.5	17.2 and 12.6	15.7
Results of EBUS-TBNA samples							
Acid-fast bacilli smear	Negative	Negative	Negative	Negative	Negative	Negative	Negative
Acid-fast bacilli culture	Negative	Negative	Negative	Negative	Negative	Negative	Negative
TB-PCR	Not detected	MTB detected	MTB detected	MTB detected	Not detected	Not detected	MTB detected
Histological grade	IV	IV	IV	IV	V	V	V
Mediastinoscopy	Performed	Not performed	Not performed	Not performed	Performed	Not performed	Not performed
Final diagnosis	Sarcoidosis	TBL	TBL	TBL	TBL	Sarcoidosis^b^	TBL

^a^Lymph nodes with the shortest diameter ≥ 10 mm on an axial CT scan

^b^Patient 6 was clinically diagnosed with sarcoidosis based on radiological and laboratory results after more than 12 months of follow-up

LN, lymph node; EBUS-TBNA, endobronchial ultrasound-guided transbronchial needle aspiration; TB-PCR; polymerase chain reaction for *Mycobacterium tuberculosis*; MTB, *Mycobacterium tuberculosis*; TBL, tuberculous lymphadenitis

### Diagnostic performance of TB-PCR

Nested PCR and real-time PCR were performed in 29 and 17 patients, respectively. TB-PCR was not detected in all patients with sarcoidosis. Of 16 patients with tuberculous lymphadenitis, nine showed positive TB-PCR results. The sensitivity, specificity, positive predictive value, and negative predictive value were 56 % (95 % CI [confidence interval], 29.9–81.2), 100 % (95 % CI, 88.3–100.0), 100 % (95 % CI, 66.2–100.0), and 81 % (95 % CI, 64.8–92.0), respectively. There was no difference in the diagnostic performance between nested and real-time TB-PCR. In addition, the overall diagnostic accuracy of TB-PCR for tuberculous lymphadenitis was 85 % in patients with IGL.

### Classification of histology

Histological results were grades I to III in 39 patients (85 %) and grades IV to V in seven patients (15 %) (Table 3). Four (9 %) and three (7 %) patients with grade IV and V disease, respectively, regarded as non-diagnostic, also showed non-diagnostic microbiological results of acid-fast bacilli smear and culture. Two patients who underwent mediastinoscopy were eventually diagnosed with sarcoidosis and tuberculous lymphadenitis (Patients 1 and 5, respectively, Table 2). Four (57 %) of seven patients with grades IV to V disease had positive TB-PCR results. All four patients achieved clinical and radiological improvement after anti-tuberculosis treatment (Patients 2, 3, 4, and 7 in Table 2).

**Table 3:** Results of histological examinations

Grade	Results of polymerase chain reaction for *Mycobacterium tuberculosis*	
	Sarcoidosis	Tuberculous lymphadenitis
I	0/0 (0)	5/8 (63)
II	0/28 (0)	0/1 (0)
III	0/0 (0)	0/2 (0)
IV	0/1 (0)	3/3 (100)
V	0/1 (0)	1/2 (50)
Total	0/30 (0)	9/16 (56)

Data are presented as the number of positive results/number of patients who received a test (%)

### Diagnostic yield of EBUS-TBNA

The overall diagnostic yield of the combined histological and microbiological data was 85 %. When the results of TB-PCR were combined with the histological and microbiological data, the overall diagnostic yield increased to 94 %.

## Discussion

In addition to acid-fast bacilli smear and culture, TB-PCR using sputum specimens is traditionally recognized as a useful examination in the diagnosis of pulmonary tuberculosis. Similar to pulmonary tuberculosis, TB-PCR using fine needle aspiration samples of cervical, axillary, or inguinal lymph nodes has also been reported as a useful molecular test [16, 17]. In the present study, we found that the sensitivity, specificity, positive predictive value, negative predictive value, and overall diagnostic accuracy of TB-PCR using EBUS-TBNA samples in patients with IGL were 56, 100, 100, 81, and 85 %, respectively. Additionally, when we compared the diagnostic yield of combined histological and microbiological examinations using EBUS-TBNA samples with a combination of the three modalities of TB-PCR, histology, and microbiology, the diagnostic yield increased from 85 to 94 % in patients with IGL. The results of the current study are in line with those of previous reports regarding TB-PCR with lymph nodes other than mediastinal IGLs.

The most important role of EBUS-TBNA in patients with mediastinal lymphadenopathy is to prevent mediastinoscopy with general anesthesia. A previous study of isolated mediastinal lymphadenopathy reported that EBUS-TBNA prevented 87 % of mediastinoscopies [7]. In the present study, seven patients had non-diagnostic EBUS-TBNA results (grade IV or V). Of them, TB-PCR was detected in four patients. In these four patients, anti-tuberculosis treatment led to clinical and radiological improvement. Therefore, the TB-PCR test could be effective and prevent mediastinoscopy when EBUS-TBNA results are non-diagnostic. Our findings suggest that TB-PCR using EBUS-TBNA samples could extend the role of EBUS-TBNA to prevent mediastinoscopy.

In a previous study of tuberculous lymphadenitis by Navani et al. [8], the diagnostic yield of combined histological and microbiological tests using EBUS-TBNA samples was much higher than that in the present study; the sensitivity was 94 %, and *M tuberculosis* was cultured in 47 % of samples. Histological and microbiological tests using EBUS-TBNA samples appear to be sufficient for the diagnosis of tuberculous lymphadenitis. However, the lymph node size examined by EBUS-TBNA in previous studies was larger than that in the current study (median, 22.0 vs. 20.0 mm, respectively). The bioburden of *M. tuberculosis* in tuberculous lymphadenitis might have been much higher in the previous study by Navani et al. compared with the current study [8].

Previous studies applied TB-PCR using EBUS-TBNA samples as follows. Geake et al. reported that the sensitivity and specificity of TB-PCR using EBUS-TBNA samples were 38 and 100 %, respectively [18]. However, TB-PCR was performed in 29 of 39 patients with tuberculous lymphadenitis, and thus selection bias might have influenced their results. Senturk et al. analyzed 30 patients who were diagnosed with tuberculous lymphadenitis (27 patients with EBUS-TBNA samples and three with mediastinoscopic samples) [19]. Analysis of TB-PCR was performed with a sensitivity of 57 % and specificity of 100 %. Generally, tissue specimens collected by mediastinoscopy are larger than those collected by EBUS-TBNA. Therefore, the sensitivity and specificity of TB-PCR presented by Senturk et al. could not be used to interpret the results of TB-PCR using EBUS-TBNA samples. Dhasmana et al. analyzed the performance of Xpert MTB/RIF in the diagnosis of mediastinal tuberculous lymphadenitis [20]. They showed that Xpert MTB/RIF had a higher sensitivity (73 %) than that found in our study (56 %). Dhasmana et al. performed Xpert MTB/RIF in patients with culture-positive tuberculous lymphadenitis. However, our study population consisted of culture-negative cases with clinical suspicion, as well as culture-positive tuberculous lymphadenitis. Not all mediastinal tuberculous lymphadenitis samples would have shown a positive culture for *M. tuberculosis*. Therefore, Dhasmana et al.*’s* results might have overestimated the diagnostic performance of Xpert MTB/RIF.

There are several limitations in this study. First, the present study was performed retrospectively. Although the data were collected prospectively, TB-PCR was not performed in four of the 50 selected patients with IGL (Fig. 1). We acknowledge that there was potential for selection bias, which might have influenced our results. Second, our study included a relatively small sample size. Third, we could not unify the PCR methods. To date, which method of TB-PCR is superior for detection of *M. tuberculosis* remains unknown. These limitations need to be verified in prospective trials with larger study populations and a uniform study protocol.

## Conclusions

TB-PCR using EBUS-TBNA samples is a useful laboratory test for diagnosing IGL. Moreover, this method can prevent further invasive evaluation in patients whose histological and microbiological tests are non-diagnostic.

## Abbreviations

CI: confidence interval
EBUS-TBNA: endobronchial ultrasound-guided transbronchial needle aspiration
IGL: intrathoracic granulomatous lymphadenopathy
TB-PCR: polymerase chain reaction for *Mycobacterium tuberculosis*

## References

[1] Statement on sarcoidosis. Joint Statement of the American Thoracic Society (ATS), the European Respiratory Society (ERS) and the World Association of Sarcoidosis and Other Granulomatous Disorders (WASOG) adopted by the ATS Board of Directors and by the ERS Executive Committee, February 1999. Am J Respir Crit Care Med. 1999;160:736–55. 10.1164/ajrccm.160.2.ats4-99 10430755

[2] Im JG, Song KS, Kang HS, Park JH, Yeon KM, Han MC (1987). Mediastinal tuberculous lymphadenitis: CT manifestations. Radiology..

[3] Iannuzzi MC, Rybicki BA, Teirstein AS (2007). Sarcoidosis. N Engl J Med..

[4] Porte H, Roumilhac D, Eraldi L, Cordonnier C, Puech P, Wurtz A (1998). The role of mediastinoscopy in the diagnosis of mediastinal lymphadenopathy. Eur J Cardiothorac Surg..

[5] Wang KP, Fuenning C, Johns CJ, Terry PB (1989). Flexible transbronchial needle aspiration for the diagnosis of sarcoidosis. Ann Otol Rhinol Laryngol..

[6] Hunninghake GW, Costabel U, Ando M, Baughman R, Cordier JF, du Bois R (1999). ATS/ERS/WASOG statement on sarcoidosis. American Thoracic Society/European Respiratory Society/World Association of Sarcoidosis and other Granulomatous Disorders. Sarcoidosis Vasc Diffuse Lung Dis.

[7] Navani N, Lawrence DR, Kolvekar S, Hayward M, McAsey D, Kocjan G (2012). Endobronchial ultrasound-guided transbronchial needle aspiration prevents mediastinoscopies in the diagnosis of isolated mediastinal lymphadenopathy: a prospective trial. Am J Respir Crit Care Med..

[8] Navani N, Molyneaux PL, Breen RA, Connell DW, Jepson A, Nankivell M (2011). Utility of endobronchial ultrasound-guided transbronchial needle aspiration in patients with tuberculous intrathoracic lymphadenopathy: a multicentre study. Thorax..

[9] Sun J, Teng J, Yang H, Li Z, Zhang J, Zhao H (2013). Endobronchial ultrasound-guided transbronchial needle aspiration in diagnosing intrathoracic tuberculosis. Ann Thorac Surg..

[10] Zhou Y, Li HP, Li QH, Zheng H, Zhang RX, Chen G (2008). Differentiation of sarcoidosis from tuberculosis using real-time PCR assay for the detection and quantification of Mycobacterium tuberculosis. Sarcoidosis Vasc Diffuse Lung Dis..

[11] World Health Organisation. Tuberculosis country profile. http://www.who.int/tb/country/data/profiles/en. Accessed 5 Nov 2014.

[12] Rusch VW, Asamura H, Watanabe H, Giroux DJ, Rami-Porta R, Goldstraw P (2009). The IASLC lung cancer staging project: a proposal for a new international lymph node map in the forthcoming seventh edition of the TNM classification for lung cancer. J Thorac Oncol..

[13] Jeong YJ, Lee KS (2008). Pulmonary tuberculosis: up-to-date imaging and management. AJR Am J Roentgenol..

[14] Bezabih M, Mariam DW, Selassie SG (2002). Fine needle aspiration cytology of suspected tuberculous lymphadenitis. Cytopathology..

[15] Alberg AJ, Park JW, Hager BW, Brock MV, Diener-West M (2004). The use of "overall accuracy" to evaluate the validity of screening or diagnostic tests. J Gen Intern Med..

[16] Aljafari AS, Khalil EA, Elsiddig KE, El Hag IA, Ibrahim ME, Elsafi ME (2004). Diagnosis of tuberculous lymphadenitis by FNAC, microbiological methods and PCR: a comparative study. Cytopathology..

[17] Pahwa R, Hedau S, Jain S, Jain N, Arora VM, Kumar N (2005). Assessment of possible tuberculous lymphadenopathy by PCR compared to non-molecular methods. J Med Microbiol..

[18] Geake J, Hammerschlag G, Nguyen P, Wallbridge P, Jenkin GA, Korman TM (2015). Utility of EBUS-TBNA for diagnosis of mediastinal tuberculous lymphadenitis: a multicentre Australian experience. J Thorac Dis..

[19] Senturk A, Arguder E, Hezer H, Babaoglu E, Kilic H, Karalezli A (2014). Rapid diagnosis of mediastinal tuberculosis with polymerase chain reaction evaluation of aspirated material taken by endobronchial ultrasound-guided transbronchial needle aspiration. J Investig Med..

[20] Dhasmana DJ, Ross C, Bradley CJ, Connell DW, George PM, Singanayagam A (2014). Performance of Xpert MTB/RIF in the diagnosis of tuberculous mediastinal lymphadenopathy by endobronchial ultrasound. Ann Am Thorac Soc..

